# Transplantation of Human Embryonic Stem Cell-Derived Retinal Tissue in the Subretinal Space of the Cat Eye

**DOI:** 10.1089/scd.2019.0090

**Published:** 2019-08-23

**Authors:** Ratnesh K. Singh, Laurence M. Occelli, Francois Binette, Simon M. Petersen-Jones, Igor O. Nasonkin

**Affiliations:** ^1^ BioTime, Inc., Alameda, California.; ^2^ Department of Small Animal Clinical Sciences, College of Veterinary Medicine, Michigan State University, East Lasing, Michigan.

**Keywords:** retinal organoids, human embryonic stem cells, subretinal transplantation, synaptic connectivity, large-eye animal models, vision restoration

## Abstract

To develop biological approaches to restore vision, we developed a method of transplanting stem cell-derived retinal tissue into the subretinal space of a large-eye animal model (cat). Human embryonic stem cells (hESC) were differentiated to retinal organoids in a dish. hESC-derived retinal tissue was introduced into the subretinal space of wild-type cats following a pars plana vitrectomy. The cats were systemically immunosuppressed with either prednisolone or prednisolone plus cyclosporine A. The eyes were examined by fundoscopy and spectral-domain optical coherence tomography imaging for adverse effects due to the presence of the subretinal grafts. Immunohistochemistry was done with antibodies to retinal and human markers to delineate graft survival, differentiation, and integration into cat retina. We successfully delivered hESC-derived retinal tissue into the subretinal space of the cat eye. We observed strong infiltration of immune cells in the graft and surrounding tissue in the cats treated with prednisolone. In contrast, we showed better survival and low immune response to the graft in cats treated with prednisolone plus cyclosporine A. Immunohistochemistry with antibodies (STEM121, CALB2, DCX, and SMI-312) revealed large number of graft-derived fibers connecting the graft and the host. We also show presence of human-specific synaptophysin puncta in the cat retina. This work demonstrates feasibility of engrafting hESC-derived retinal tissue into the subretinal space of large-eye animal models. Transplanting retinal tissue in degenerating cat retina will enable rapid development of preclinical in vivo work focused on vision restoration.

## Introduction

Vision is by far the most dominant and most important sense to all primates, including humans, with 80% or more of all sensory information being perceived by means of sight [1–4]. Loss of vision is very debilitating and costly to patients, their families, and health care [5,6]. Retinal degenerative diseases, which include conditions such as age-related macular degeneration and retinitis pigmentosa (RP), are a major cause of blindness, affecting people worldwide [7–12]. At present, there is no satisfactory treatment available to restore vision following photoreceptor (PR) death, for patients with these blinding conditions, highlighting the fact that vision restoration is a major unmet need and a major medical challenge. Therefore, new and effective treatments to restore and preserve vision in patients with retinal degeneration (RD) are urgently needed. Previous tissue restorative studies have focused on using human fetal retinal tissue for replacement [13–19] and stem cell-based therapies focused on replacing PRs [8,20–24]. There are issues with and limitations to both approaches [13,15,22,25–31]. However, there is a clear similarity between grafted retinal sheets and some successful gene augmentation therapies of RP and Leber congenital amaurosis models, where the treated retinal areas remain as surviving patches of retina [32], which maintain visual function [33–36]. Similar to patches of retina preserved by gene augmentation therapy, the islands of transplanted mutation-free retina have the potential to survive, and with synaptic connectivity to the host, at least partially restore visual function [15,16,18,19,37–40]. On the contrary, the progressive nature of RD conditions, where the dying PRs destroy RD retinal matrix and trigger the death of healthy PR around them [41–45], indicates that transplanting dissociated mutation-free retinal cells into the degenerating retinal milieu is a challenging approach, at least for rapidly progressing RD conditions. Furthermore, functional cell replacement is a complex task because the new cells must migrate to specific locations in the retinal layers and re-establish specific synaptic connectivity with the host. Synaptic remodeling of neural circuits during advanced RD further complicates this task [46–49]. Restoring the original neural architecture of the retina, undergoing advanced RD, may be a difficult task due to degenerating retinal milieu, loss of cells, and distorted neuroanatomy, exacerbated by progressive remodeling [46]. Using fetal retinal tissue to provide a graft to treat patients is ethically challenging and the availability of tissue is limited [50]. The use of organoid tissue grown in vitro from approved human embryonic cell lines overcomes these ethical concerns and limited tissue availability. To practically restore at least useful vision and ameliorate blindness caused by RD conditions (a major translational goal [51]), new and realistic ideas are urgently needed, which take advantage of new technologies and approaches.

Advances in regenerative medicine enabled generation of three-dimensional tissues (organoids) [52–56], partially recreating the anatomical structure, biological complexity, and physiology of several tissues, which are important targets for stem cell replacement therapies. Derivation of retinal tissue in a dish from human embryonic stem cells (hESCs) and human-induced pluripotent stem cells (hiPSCs) creates new opportunities for designing tissue replacement therapies for blindness and addresses the need to preserve retinal architecture to restore vision. Moreover, this approach can utilize the 30-year experience and knowledge of transplanting sheets of human fetal retina [13,57]. It is also realistic as it is already revealing signs of clinical promise in animal models. Transplantation of iPSC-derived retinal tissue in murine RD models has demonstrated visual improvement [58,59]. Transplanted iPSC-retina developed outer nuclear layer (ONL) with mature outer segments and synaptic connectivity with the host neurons. These studies provide evidence that semidifferentiated retinal tissue grafts placed into subretinal space of animals with RD undergo lamination and cell fate commitment, and establish functional connectivity between graft-derived PRs and recipient retina, as first demonstrated some 20 years ago by Aramant and Seiler [16,37]. However, therapeutic studies in small-eye animal models such as rats and mice have limitations and may not directly translate into the clinic; the rodent eyes are small compared to human eyes; they do not have maculae and have proportionally very large lens and small vitreal cavity [60], meaning that they require a different surgical approach from what would be needed in patients. In addition, small eye size makes it challenging to evaluate the size of a patch needed for therapy in a human eye to restore useful vision. In contrast, large-eye animals with inherited RD mutations such as dogs [61,62], cats [63–65], and pigs [66–68], and primates with induced RD [69–72] provide better opportunity to translate in vivo findings to blind patients. Both dogs and cats have an *area centralis* [65,73,74], which is functionally similar to primate macula. Moreover, due to the large size of their eyes, dogs and cats enable the development of surgical skills and approaches for grafting hESC-3D retinal tissue, which can be directly translated to the clinic. Shirai et al. transplanted hESC-derived retinal tissue into the immunosuppressed monkey model of RD [72]. Grafted hESC-retinal tissue underwent maturation and developed postmitotic retinal cell phenotypes, including rod and cone PRs, and formed synaptic connectivity with the host retina. Studies of Seiler and Narfström [75], also Bragadottir and Narfström [76], demonstrated the survival of fetal retinal sheets in the subretinal space of cats.

The focus of this article is to demonstrate the feasibility of grafting hESC-derived retinal tissue (retinal organoids) into the subretinal space of a large-eye animal (wild-type cat) before moving this work to RD cat model. Therefore, in this study, we addressed the following challenges: (i) developing the surgical procedure of grafting tissue in a large eye, (ii) overcoming the immune rejection of the recipient, (iii) achieving maturation of the retinal tissue, and (iv) graft-> host connectivity. The establishment of these approaches leads the way to preclinical therapeutic studies utilizing cat RD models.

## Materials and Methods

### Cell culture and retinal differentiation

The hESC line (HES03) was obtained from BioTime, Inc. The cells were maintained in feeder-free conditions using mTeSR1 [56,77] with the addition of heparin (10 ng/mL) and 1× amphotericin-B/gentamicin on Matrigel-coated plates in a 37°C incubator with lower oxygen (17%–18%). Cells were passaged every 5–6 days (reaching 80% confluency by day 7) on Matrigel-coated 35-mm plates using the Versene/EDTA (at a ratio of 1:10). Karyotype was verified by Cell Line Genetics. Neural induction of hESCs was started with noggin [8,56,78–80] when hESC colonies reached 75%–80% density. On day 0, we replaced hESC medium with hESC medium/Neurobasal complete (NB) medium (1:1 ratio) with no extra basic fibroblast growth factor (bFGF) and 100 ng/mL human noggin morphogen (Peprotech, Rocky Hill, JN), then (on day 3) replaced the medium with 100% NB with 1× N2, 1× B27, and 100 ng/mL noggin, and cultured for another 3 days [56]. We continued replacing ½ of the conditioned medium every third day with fresh NB/N2/B27/100 ng/mL noggin. At +2 weeks after initiating the protocol, we applied human bFGF (20 ng/mL; Peprotech). At +4 weeks, when neural rosettes were plentiful in differentiating 2D adherent monolayer, we applied human Dickkopf protein DKK-1 and human insulin growth factor-1 (IGF-1), 20 ng/mL each, both from Peprotech [8,56,79] for 1 week. The plates were then cultured for 3–4 weeks in Neurobasal complete medium with human noggin (100 ng/mL), also human bFGF, and human FGF9 (both at 20 ng/mL) [8,56] to promote neural retinal differentiation. Clusters of retinal differentiation (identified by growing 3D retina surrounded by areas of brown RPE cells) were manually harvested using a thin sterile Pasteur pipette (pulled over a flame to generate a flexible glass rod in the shape of a hook). The clusters were then further grown for up to several weeks in nonadherent conditions (on an orbital shaker, 40–50 rpm in low-attachment six-well plates) at 37°C/5% CO_2_ in normoxic conditions (21% oxygen), with the addition of 20 ng/mL human brain-derived neurotrophic factor (BDNF) (R&D Systems, now BioTechne, Minneapolis, MN) and 20 ng/mL human bFGF. Approximately half of media was changed two to three times/week.

Immunohistochemistry of retinal organoids was done as described earlier [56]. Briefly, the organoids were fixed in freshly prepared 4% paraformaldehyde (Electron Microscope Sciences, Hatfield, PA) in phosphate-buffered saline (PBS; Sigma-Aldrich Corp., St. Louis, MO), pH 7.8 for 30 min at room temperature, then rinsed thrice in PBS (10–20 min each, room temperature), saturated in sucrose (10% sucrose/PBS, 1 h, then 20% sucrose/PBS, 2 h, and then 30% sucrose/PBS, 2–3 h, on an orbital shaker at room temperature), embedded in Tissue-Tek optimum cutting temperature compound (Torrance, CA) (3 volumes): 30% sucrose (1 volume) in small (8 × 8 mm) cryomolds, and snap-frozen in ethanol/dry ice bath. Cryoblocks with embedded human retinal organoids were sectioned at 12 μm with a cryostat (Thermo Fisher Scientific Microm HM550–388114) at −20°C.

### Shipping of retinal organoids

Retinal organoids were stored in Hibernate E medium containing 20 ng/mL human BDNF and 20 ng/mL human glial derived neurotrophic factor (both from R&D Systems) (as described by Aramant and Seiler [15,17]) during shipment from Biotime, Inc. to Michigan State University (MSU). Hibernate E medium is specially formulated to keep embryonic tissue alive when refrigerated without oxygen or CO_2._ We extracted the temperature plots inside the incubator using the multiuse temperature probe (TempTale^®^Ultra; Sensitech, Inc.). As a test, we shipped samples of mouse embryonic retina before shipping retinal organoids in exactly the same conditions by overnight FedEx from BioTime (Alameda, CA) to MSU (East Lancing, MI) (harvesting time: 3 pm the day before, receiving time by 10 am next day), fixed on arrival with freshly prepared 4% PFA for 30 min, processed for histology, and stained with antibodies to Cleaved Caspase 3 and Gamma H2AX [81]. We did not find any signs of early apoptosis (data not shown).

### Subretinal transplantation of retinal organoids

All animal procedures were approved by the Institutional Animal Care and Use Committee of the MSU and conducted in accordance with the ARVO Statement for the Use of animals in Ophthalmic and Vision Research. The subretinal transplantation of retinal organoids was performed by a boarded veterinary ophthalmologist (S.P-J.). Cats were anesthetized with isoflurane and placed in dorsal recumbency. The eye was positioned in primary gaze and aseptically prepared for a routine two-port partial 23-gauge vitrectomy. Visualization of the posterior segment was by use of an irrigating vitrectomy lens (Machemer Vitrectomy Lens; Ocular Instruments, WA).

Sclerotomies were 5 mm posterior to the limbus. A core vitrectomy (Accurus; Alcon, Fort Worth, TX) and detachment of the posterior vitreous face over the region of planned implantation were performed and with visualization from triamcinolone crystals (Kenalog Suspension Bristol-Myers Squibb) that were previously washed in Balanced Salt Solution (BSS; Alcon). A subretinal injection of BSS was performed using a RetinaJect (RetinaJect Subretinal Cannula; SurModics, Inc., Eden Prairie, MN). The sclerotomy port was enlarged to accommodate the organoid glass injection cannula and a retinotomy performed into the detached retina with retinal scissors to allow entry of the injection cannula into the subretinal space. Organoids were kept at 37°C in 5% CO_2_-saturated tissue culture incubator in the culture medium until 10 min before implantation. Organoids were loaded into the injection cannula, a borosilicate tube 1.52 mm outer diameter (OD) and 1.12 mm inner diameter (ID) TW150–4 (World Precision Instruments, Sarasota, FL), directly in surgery room using a syringe attached to the cannula. Large organoids were cut in half (0.3–0.5 mm), while small organoids (which fit into the cannula) were transplanted as whole organoids (5–9 organoids/graft). The dish was placed on a 37°C surgical warming pad during cutting and loading steps. Organoids were injected into the subretinal space under direct visualization. Following placement of the organoids, the sclerotomies were closed using 6–0 Coated Vicryl suture (Ethicon, Inc., Somerville, NJ). The conjunctiva and lateral canthus were closed in a routine manner. At the end of the procedure, a subconjunctival injection of a mixture of 0.1 mg dexamethasone (Bimeda-MTC Animal Health, Inc., Cambridge, ON, Canada), 2 mg methylprednisolone (Zoetis, Inc., Parsippany-Troy Hills, NJ), and 1 mg gentamicin (Thermo Fisher Scientific, Waltham, MA) was performed.

Immunosuppressive therapy consisted of twice daily oral prednisolone (1 mg/kg; Hi-Tech Pharmacal Co., Inc., Amityville, NY) and in the second group of animals, also cyclosporine (7 mg/kg; Elanco Animal Health, Greenfield, IN) starting 3 days before the procedure.

### Retinal imaging

Color fundus images were captured using the RetCam II (Clarity Medical Systems, Inc., Pleasanton, CA) immediately following the transplantation and periodically thereafter. For sessions following the postoperative imaging, pupils were dilated with 1% tropicamide (Tropicamide, Akorn, Inc., Lake forest, IL) and a topical sterile anesthetic applied (Alcaine, Proparacaine hydrochloride ophthalmic solution; USP 0.5% Bausch & Lomb, Rochester, NY). Standard light conditions were used for color fundus images.

Scanning laser ophthalmoscope (cSLO) and spectral domain optical coherence tomography (SD-OCT), retinal cross-sectional images of the graft were captured (Spectralis Heidelberg Engineering, Heidelberg, Germany) under general anesthesia (mask induction with isoflurane, and intubation and maintenance on inhaled isoflurane delivered in O_2_) with the animals placed on a heating pad maintained at 37°C. A lid speculum and conjunctival stay sutures were used to maintain the globe in primary gaze. Both infrared and autofluorescent cSLO imaging were performed. High resolution line and volume scans were used to record graft and host retina appearance; enhanced depth imaging protocols were used as required.

### Preparation of cat ocular tissue

Cats (five, total of seven eyes with subretinal grafts, based on the SD-OCT imaging) were euthanized using pentobarbitone according to the AUF protocol 05/17-075-00. The eyes were enucleated, incisions were made through the pars plana and the globes immersed in 4% paraformaldehyde (Electron Microscope Sciences) in PBS (Sigma-Aldrich Corp.) on ice for 3.5 h. The anterior chambers were then removed and the vitreous removed, and the eyecups were fixed for another 10–30 min in the same fixative depending how much vitreous was left. After three washes of 10 min each in 1× PBS, the cat eyecups were cryoprotected in sucrose solutions prepared in PBS, pH 7.8 (15% sucrose for 1–2 h until sinking, and then 30% sucrose for an hour). After two washes of 5 min each in 1× PBS, the cat eyecups were then snap-frozen in optimal cutting temperature embedding material (Tissue-Tek, Sakura Finetek, Torrance, CA) in a beaker partially filled with methanol on liquid nitrogen. They were then stored at −20°C until sectioned.

### Cryosectioning and slides

The Microm HM550 cryostat (Thermo Scientific, Rockville, MD) was used to produce 16 μm serial sections of cat eyes. Microscope slides were purchased from Fisher Scientific (Pittsburg, PA). Glass coverslips were purchased from Brain Research Laboratories (Newton, MA). Eye cups were serially sectioned at 12 μm.

### Immunohistochemistry of cat ocular tissue

The sections were first permeabilized with 0.1% triton X-100/PBS (PBS-T) at room temperature for 30 min, followed by 1 h of incubation in blocking solution (5% preimmune normal goat serum; Jackson ImmunoResearch, West Grove, PA) and 0.1% PBS-T at room temperature, and then incubated with primary antibodies diluted in blocking buffer at 4°C overnight (Supplementary Table S1). The following day, sections were washed thrice (10–15 min each time) with PBS-T, and then incubated with the corresponding secondary antibody at room temperature for 45 min. The slides were washed twice with 0.1% PBS-T solution, incubated with 4′, 6-damidino-2-phenylindole (DAPI) solution (1 μg/mL) for 10 min, and then washed again with 0.1% PBS-T solution. The specimens were mounted with ProLong Gold Antifade medium (ThermoFisher Scientific) and examined using a ZEISS confocal microscope (Oberkochen, Germany).

## Results

### Differentiation of hESCs to retinal tissue

**FIG. 1.**
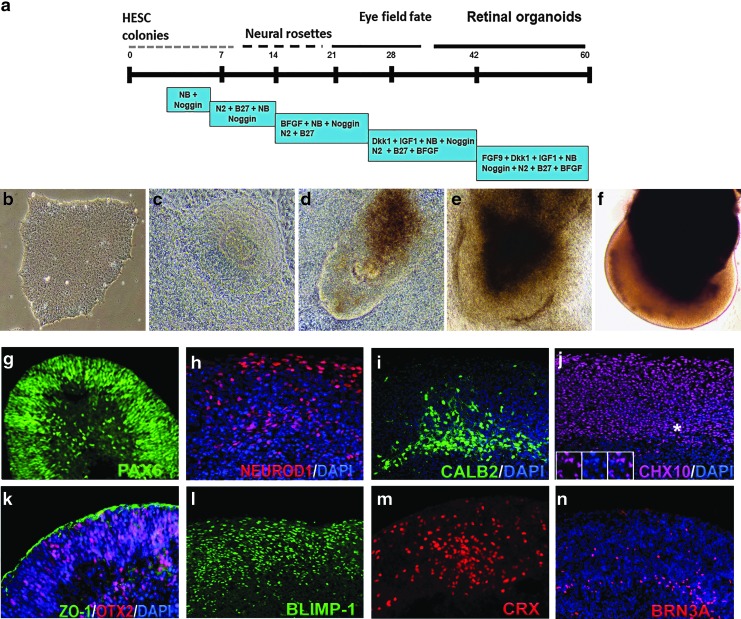
Differentiation of hESCs to retinal tissue (retinal organoids) and immunocytochemical characterization of retinal organoids before transplantation. **(a)** Schematic of three-dimensional retinal organoid differentiation protocol. **(b–f)** Representative bright-field images of retinal differentiation stages in culture. **(g–n)** Immunocytochemistry of hESC-derived retinal tissue (9–10 weeks) with antibodies specific to PAX6, NEUROD1, CALB2, CHX10, OTX2, ZO-1, BLIMP1, CRX, and BRN3A. *Insets* in panel **j** represent the magnification of the area marked with *asterisks*. hESC, human embryonic stem cell.

In our earlier work, we differentiated hESC line H1 to retinal tissue using Noggin, DKK1, and IGF1 [56]. Using a modified protocol, we could reproducibly differentiate the hESC line HES3 into retinal tissue (Fig. 1a–f). On day 50–70, we consistently observed retinal progenitor/eye field marker PAX6 [82,83], OTX2, pan-neural retina progenitor marker CHX10 (VSX2) [84–87], photoreceptor progenitor marker CRX [88,89], photoreceptors and amacrine progenitor marker NEUROD1 [90–95], photoreceptor progenitor marker BLIMP1 [96–98], amacrine marker CALB2 (Calretinin) [99–101], and retinal ganglion marker BRN3A [102] in hESC- derived retinal tissue. The retinal pigment epithelial layer was detected by immunolabeling with tight junction protein *zonula occludens* (ZO)-1 [103] and pigmented RPE marker PMEL17 [70,104] (Fig. 1g–n and Supplementary Fig. S1). Cell proliferation marker Ki67 was present on the apical side of the retinal organoids [56] (data not shown). Photoreceptor progenitors were present on the apical side of the hESC-derived retinal tissue, whereas amacrine cells were restricted to the basal side. To demonstrate our retinal differentiation protocol is efficient and does not leave any undifferentiated cells in the organoids, we immunostained retinal organoid sections and papain dissociated organoids with anti OCT3/4 antibody and CHX10 antibody (Supplementary Fig. S2). We did not find cells carrying pluripotent markers. However, we found abundant presence of CHX10-positive cells. HES3 colonies were used as OCT3/4 positive control.

### Transplantation of hESC-derived retinal tissue in subretinal space of wild-type cats

**FIG. 2.**
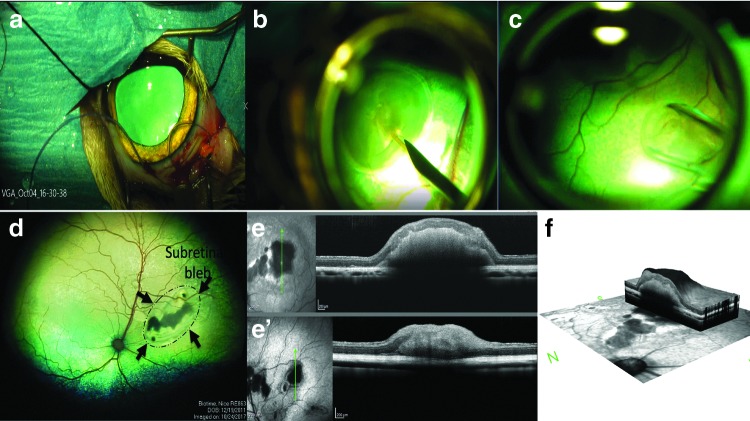
Transplantation of hESC-derived retinal tissue into the subretinal space of wild-type cats and imaging of grafts. **(a)** A routine two-port partial 23-gauge vitrectomy (following lateral canthotomy and conjunctival peritomy) is performed. **(b)** Creating subretinal bleb using Balanced Salt Solution delivered by a RetinaJect subretinal injection cannula. **(c)** Organoids were loaded into the glass cannula using a syringe attached to the cannula. **(d)** Retinal organoids can be seen in the subretinal space (RetCam II imaging). *Black arrows* indicate the extent of the retinal bleb that was formed before subretinal transplantation. **(e–f)** SD-OCT images showing presence of grafts in the subretinal space. SD-OCT, spectral-domain optical coherence tomography.

HESC- derived retinal tissue differentiation, day (DD) 60–70, was implanted into the subretinal space of five adult cats (Table 1 and Fig. 2; Supplementary Fig. S3). The initial pilot study with two cats (three grafts) was performed to work out transplantation procedure and see if prednisolone alone would be sufficient to prevent graft rejection. In subject 1, implantation was only successful in the left eye. This cat was maintained for 28 days (Table 1). In subject 2, we implanted retinal organoids in both eyes and the cat was maintained for 66 days (Table 1). No evidence of graft-related inflammation was detected by ophthalmoscopy. The grafts were visualized in the subretinal space by ophthalmoscopy and SD-OCT.

**Table 1.** Details of Cat Subretinal Implantation

*Animal (Cat)*	*Age*	*Sex*	*Eye*	*Result of surgical transplantation*	*SD-OCT findings*	*Duration*	*Immuno suppression*	*Notes*
Subject 1	5.7 years	F	OD	Unsuccessful	—	28 days	Prednisolone only	
			OS	5 organoids implanted subretinally	Organoids remained separate. Some disruption of overlying retinal lamination.			Poor survival, medium-size graft, massive number of Iba1[+] cells
Subject 2	5.7 years	F	OD	5 organoids implanted subretinally	Organoids present in subretinal space. Lamination of overlying retina preserved	66 days	Prednisolone only	Poor survival, large graft, patches of surviving HNu[+] cells remaining, massive number of Iba1[+] cells
			OS	5 organoids implanted subretinally	Organoids present in subretinal space. Lamination of overlying retina preserved			Poor survival, very small patch of HNu[+] cells remaining
Subject 3	7.4 years	F	OD	Unsuccessful	—	36 days	Cyclosporine A and prednisolone	
			OS	8 organoids implanted subretinally	Organoids appeared to coalesce into one structure. Some disruption of overlying retinal lamination.			Good survival of human graft
Subject 4	6 years	M	OD	6 organoids implanted subretinally	Organoids appeared to coalesce into one structure. Some disruption of overlying retinal lamination	36 days	Cyclosporine A and prednisolone	Good survival of human graft
			OS	9 organoids implanted subretinally	Organoids appeared to coalesce into one structure. Some disruption of overlying retinal lamination.			Cellular infiltration in peripheral residual vitreous graft did not survive
Subject 5	6 years	F	OD	Unsuccessful	—	36 days	Cyclosporine A and prednisolone	
			OS	8 organoids implanted subretinally	Organoids appeared to coalesce into one structure. Some disruption of overlying retinal lamination.			Good survival of human graft

OS, left eye; OD, right eye.

In the second cohort of cats (subjects 3–5), we transplanted retinal organoids in subretinal space and maintained the cats for 36 days, while treating with a combination of cyclosporine A and prednisolone (Table 1). Immunosuppression was provided starting 3 days before implantation and maintained for the duration of the study. In two cats, subretinal transplantation of organoids was successful in one eye and in one cat, bilateral transplantation was achieved. In one eye of subject 5, a cellular infiltration into the remaining vitreous developed. No obvious indicators of inflammation were seen in the other three eyes that had successful transplants.

SD-OCT examination was performed on all transplanted eyes (Fig. 2e, f and Supplementary Fig. S3). The retinotomy could be identified, but despite a relatively large retinotomy required for introduction of the capillary tube delivering the organoids, the retina reattached in all eyes. The retina adjacent to larger organoids was separated from the RPE due to the thickness of the organoid, but was otherwise not detached. In some instances, separate organoids could be discerned, but very often, the separate organoids appeared to have coalesced into a single structure. Varying degrees of altered lamination of the overlying host retina was present, but in most instances, the main retinal layers were present.

### Survival of retinal organoid in subretinal space of wild-type cats with prednisolone and cyclosporine A

We first used pilot cohort (prednisolone only) to evaluate the extent of graft survival.

**FIG. 3.**
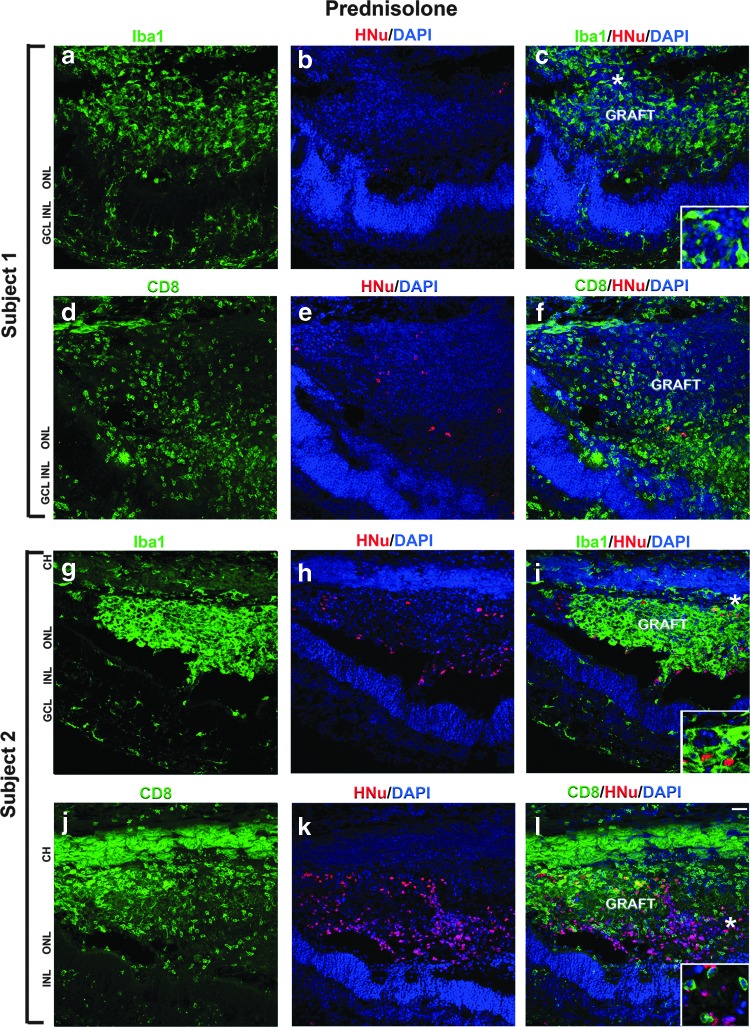
Infiltration of Iba1- and CD8-positive cells in the subretinal grafts maintained with prednisolone immunosuppression. **(a–l)** In subjects 1 and 2, we observed many Iba1-positive and CD8-positive cells in the grafts and surrounding host tissue. *Insets* represent the magnification of the area marked with *asterisks*. HNu staining (*red*) shows the poor survival of grafts. Scale bar: 50 μm. CH, choroid; ONL, outer nuclear layer; INL, inner nuclear layer; GCL, ganglion cell layer.

In subject 1, the SD-OCT image showed small- to medium-sized graft, whereas in subject 2, one of the two grafts was large. Therefore, we euthanized subject 1 at 28 days, whereas subject 2 was euthanized at 66 days. Presence of grafts in the subretinal space of subjects 1 and 2 was detected by immunostaining with antibodies specific to human markers HNu (human nuclei) [78] and Ku80 (human nuclei) [59] [78,105]. Interestingly, we observed either absence of HNu staining (subject 1) or diffused staining (subject 2) in the grafts (Fig. 3). In subject 2, we also observed some isolated areas where HNu- and Ku80-positive nuclei were preserved. However, these small patches of human nuclei were surrounded with large areas where nuclei had altered and fragmented morphology (Fig. 3k and Supplementary Fig. S4a–d).

Next, we determined the infiltration of inflammatory or immunogenic cells in these grafts. Inflammatory and local immune response were detected by staining the retinal section with anti-ionized calcium-binding adaptor molecule 1 (Iba1) (also known as Allograft Inflammatory Factor, AIF-1) [8,106,107] and cytotoxic T-lymphocyte (CTL) cell marker CD8 [108,109], respectively. In both subjects 1 and 2, we observed acute graft rejection. Iba1-positive cells (ameboid microglia and macrophage) invaded the grafts and were also present in the choroid and host retina (Fig. 3; Supplementary Figs. S4 and S5), indicating the inflammatory response to the grafts. Anti-CD8 antibody to activated T cell marker of CTL staining demonstrates strong presence of CD8-positive cells in the grafts and in the choroid, and in host retina surrounding the grafts (Fig. 3).

**FIG. 4.**
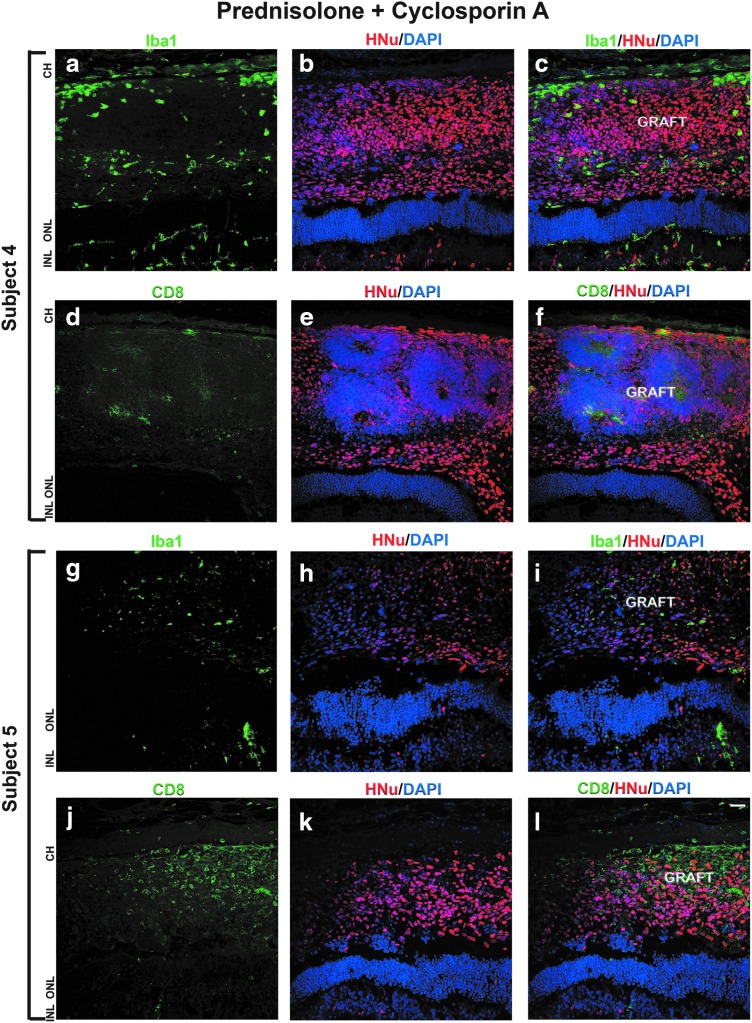
Mild immune response to the grafts maintained with prednisolone + cyclosporine A immunosuppression. **(a–l)** In subjects 4 and 5, we observed few Iba1- and CD8-positive cells in the subretinal grafts and surrounding host tissue. HNu staining shows good survival of graft.

On the contrary, we observed better graft survival and mild immune response in the subjects 3, 4, and 5, which were treated with a combination of prednisolone and cyclosporine A immunosuppression regimen (Fig. 4).

Immunostaining with anti-HNu antibody revealed well-defined nuclei with no evidence of nuclei fragmentation. Immunostaining with anti-Iba1 antibody showed low infiltration of Iba1-positive cells in the graft and surrounding area. The number of CD8-positive cells in the graft and surrounding cat retina was low (Fig. 4). We observed similar results in all the subjects 3, 4, and 5. Immunostaining the sections with antibody to cell proliferation marker Ki67 showed presence of some Ki67-positive cells in the grafts (data not shown).

At the time of transplantation, the retinal organoids had only retinal progenitors and RPE markers. After 36 day of post-transplantation, we also found BRN3A, CHX10 [82,110], CRX, CALB2 [101], synaptophysin (SYP), gamma*-*aminobutyric acid (GABA), and PMEL17 in the grafts (Supplementary Figs. S6, S7, S8, S9, S10). Interestingly, we observed some BRN3A-, CHX10-, and CALB2-positive cells migrating from the subretinal space to the host ONL, inner nuclear layer (INL), and retinal ganglion cell (RGC) layer. Similarly, we also found PMEL17-positive cells migrating toward the host RPE layer. We did not observe any mature photoreceptor marker such as rhodopsin and peripherin in the grafts. Together, these results indicate better survival of grafts and reduced immune response in the subretinal space of the eyes treated with both prednisolone and cyclosporine A.

### Extension of axonal and synaptic connectivity between the graft and host retina

**FIG. 5.**
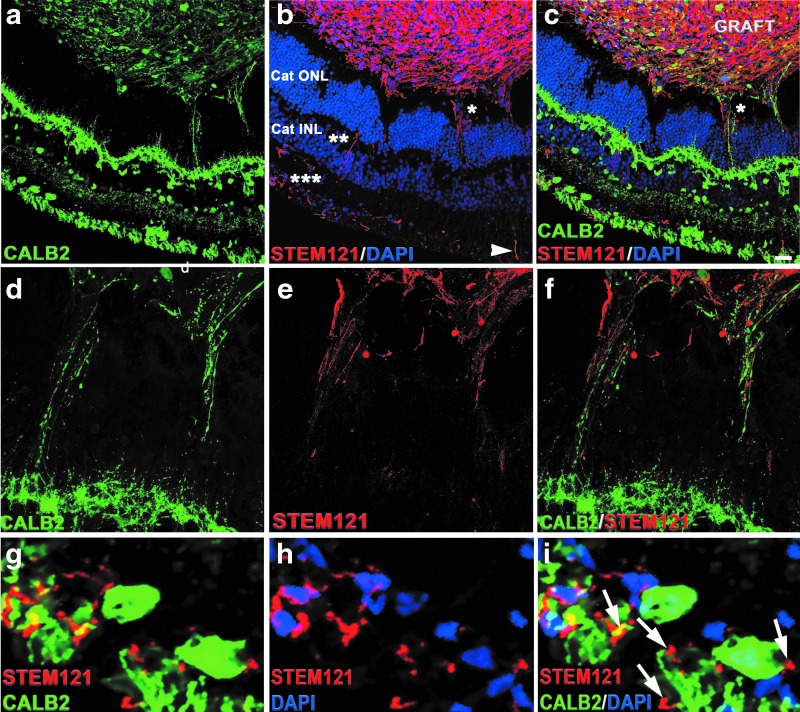
Cytoplasmic projections connecting the graft and the host tissue. **(a)** Immunohistochemical staining shows presence of CALB2 in the graft and the host tissue. **(b)** STEM121 staining was restricted to the grafts. In addition, we observe STEM121-positive projections emanating from the graft to the host ONL (*), INL (**), and RGC layers (***). **(c)** Co-immunostaining of cat sections with CALB2 and STEM121 shows the cytoplasmic projections are not co-localized. **(d–f)** High magnification of the area marked with an *asterisk* (*) marked in **(b)** shows the cytoplasmic projections positive for CALB2 and STEM121 do not colocalize. **(g–i)** High magnification of the area marked with triple *asterisks* (***) shown in **(b)**. *Arrows* indicate the STEM121-positive projections contacting the cat RGC layer. Scale bar: 50 μm. RGC, retinal ganglion cell.

To determine the potential axonal and synaptic connectivity between the graft and the host, we used antibodies specific for human cytoplasm (STEM121) [78], human-SYP (presynaptic part of human synapses) [78], and CALB2 [101]. We observed strong presence of calretinin-positive and STEM121-positive cells in the grafts. Calretinin immunostaining was present in the host outer plexiform layer and inner plexiform layer [46,101]. We found calretinin-positive fibers connecting graft and the host. However, STEM121 staining was restricted to graft cytoplasm. We observed large number of STEM121-positive fibers projecting from the graft toward the host ONL, INL, and RGC layers. Interestingly, these STEM121-positive fibers were passing through the cat ONL and ending up in cat INL and in the cat RGC layer (Fig. 5b–i and) [101]. The majority of CALB2-positive fibers connecting the INL and the grafts were STEM121 negative. We also observed some CALB2-positive and GABA-positive projections emanating from the graft to the host (Supplementary Fig. S10).

To investigate whether these cytoplasmic projections represented young axons projecting from the graft, we co-stained sections with antibody to STEM121 and the antibody to doublecortin (DCX), and performed high-magnification confocal analysis with z-stacking and compression of z-stack, to better visualize the projections in sections. DCX is a marker of early stages of axonal formation [111,112]. We indeed observed colocalization of STEM121 and DCX in some, but not all STEM121-positive fibers (Supplementary Fig. S11). To gain additional insight about the nature of these projections, we co-stained the sections with antibodies to human nuclei (Ku80) and pan-axonal cocktail of antibodies SMI-312. We observed strong SMI-312-positive staining in the grafts. In addition, we found SMI-312-positive fibers connecting the graft and the host (Supplementary Fig. S12). Collectively, these results indicate that the fibers connecting the cat retina and the grafts are young pathfinding axons.

To determine if STEM121-positive fibers found in the GCL migrate toward the optic nerve, we immunostained cat retina with NF200 (axonal marker) and STEM121 antibodies. We found few STEM121-positive fibers running parallel to the host RGC layer (Supplementary Fig. S13). Interestingly, STEM121-positive fibers were negative for NF200. Further immunostaining the cat optic nerve head with STEM121, DCX and NF200 revealed few STEM 121-positive fibers in the optic nerve, which were also positive for DCX (Supplementary Fig. S13).

**FIG. 6.**
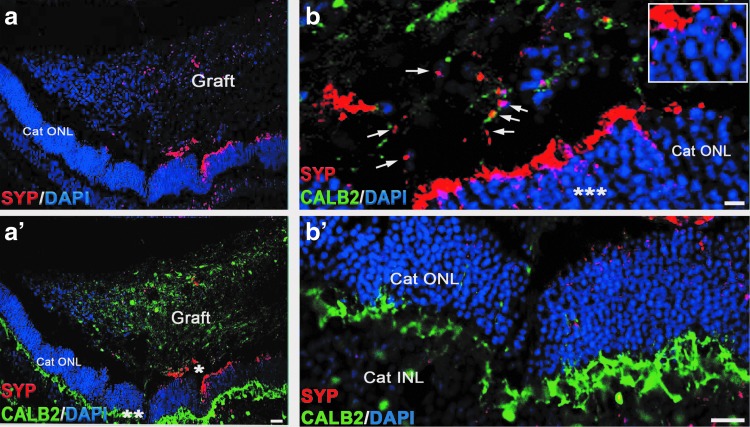
Synaptic interaction between the graft and the host tissue. Low magnification images demonstrate presence of SYP staining in the graft and in cat ONL adjacent to the graft. **(a-a’)** Shows co-labeling of cat retinal section immunostained with SYP and CALB2. **(b-b’)** High magnification images of the area marked with *asterisks* (*) and (**) in **(a’)**, showing SYP-positive boutons in the graft and host ONL. *Arrows* indicate SYP-positive boutons in the graft. The *inset* in **(b)** is a high magnification of area marked with *asterisk* (***). Scale bar: 20 μm **(b)**; 50 μm **(b’).** SYP, synaptophysin.

To determine if STEM121-positive fibers may be developing synapses with host neurons, we stained sections with antibody to human SYP, which can robustly decorate the presynaptic part of human (but not cat, rat, or mouse) synapse [59,78]. We observed patches of SYP-positive puncta in the grafts and in the cat ONL and INL/IPL (Fig. 6).

## Discussion

In this article, we outline the surgical procedures for grafting hESC-derived retinal tissue from organoids into the subretinal space of the cat eye, an immunosuppression protocol to allow graft survival, and the very promising outcomes of grafting, demonstrating robust survival and axonal connectivity (graft to host and host to graft). This work lays the foundation for developing retinal and vision restoration technologies in a large-eye model, relevant to human therapies.

There are suitable spontaneous genetic models of early-onset RD in the cat, such as the *Crx^+/−^* [65] and *Aipl1^−/−^* cat [64], making it a very good model for developing cell therapies in the ocular space and specifically therapies focused on RD. Cats have cone-rich *area centralis*, which is analogous to human maculae. The size of an adult cat's eye is very similar to the size of adult human eye [74], enabling easy translation of surgical techniques to patients.

In this study, we chose to transplant retinal organoids at DD 60–70, which is in line with the work of others [59,113]. If hESC-derived neural grafts are too immature, they tend to have high mitotic index [78], and conversely, if they are transplanted late, they may not integrate with the host and/or die [13] (IN, unpublished observation).

Initially, we chose to test a mild immunosuppression regimen (prednisolone only) because the subretinal space is an immune-privileged site [55,114–117], and sustained release of corticosteroids, including prednisolone, has been discussed and noted as a potent suppressor of intraocular inflammation [118,119].

Although the grafts in the first cohort of cats were shown on IHC to have undergone rejection, the eyes did not show any gross evidence of inflammation. Two of the three grafts in this group decreased markedly in size, highlighting the rapid death of the human grafts in the xenogenic conditions, while the third graft was larger and retained patches of surviving HNu-positive cells. Interestingly, although the grafts in the prednisolone-only group could be seen by SD-OCT, funduscopy, and cSLO, the IHC data demonstrated very poor-to-no survival of human cells in grafts, and the pronounced presence of Iba1-positive microglia. CD8-positive infiltration was also pronounced in these grafts. We have previously observed similar results when describing the survival of the grafts of hESC-derived retinal progenitors in rodent work (also a xenograph). Specifically, while some subretinal grafts were clearly visible by histology as a bulge, filled with cresyl violet-positive cells, such grafts had no, or only few, HNu-positive cells, but were filled with host-specific Iba1-positive microglia [8]. In subject 2, the graft size was larger. Therefore, we expected that the cells may survive longer (should prednisolone be insufficient for immunosuppression), making this graft at least more informative. In agreement with our initial findings in subject 1, we observed poor survival of the graft in subject 2 (Fig. 3; Supplementary Figs. S4 and S5) and many Iba1-positive and CD8-positive cells in and around the graft site. This is in line with our earlier observation [8] and may be due to the breach of blood-retinal barrier during retinotomy.

Some of the difficult-to-explain differences in the efficiency of survival of human retinal cells in the xenogeneic grafts (even in the presence of immunosuppression) may be related to the animal model used rather than cells implanted. For example, if rabbits (which have merangiotic retina) are used [120], the retinotomy can be positioned to avoid retinal vasculature, reducing the contribution of transplantation site bleeding to the development of graft rejection.

Other reported cases of graft rejection [70,121] can be related to the impact of innate immunity [122] and/or surgical complications, for example, bleeding [8], which are likely to be related. Although work in animal models places human grafts in a more challenging environment (as they are xenogeneic grafts) than would be expected in the actual clinical settings (where they will be allogeneic grafts), this preclinical work demonstrates potential pitfalls of cell therapies and will enable the development of fail-proof protocol and directions, which will ultimately robustly work in the subretinal space to restore vision. One approach in patients may be the partial major histocompatibility (MHC) matching of donor cells to the MHC profile of a recipient [123]. Yet another critical and recurrent aspect of this work is the development of better surgical methods to avoid activating the innate immunity of the recipient and causing a breach in the blood-retinal barrier [8]. In this study, we found that prednisolone alone was clearly not sufficient to allow survival of the xenogeneic human graft in the cat subretinal space.

In the second cohort of animals, we used a stronger immunosuppressive regimen, combining cyclosporine A with prednisolone [27,124], to specifically inhibit the CD8-positive CTLs [125–127]. We found robust survival of the grafts in subjects 3, 4, and 5 in prednisone + cyclosporine A-immunosuppressive conditions. In one eye of subject 5, there was a marked cellular infiltration into the vitreous, which most likely represents an endophthalmitis due to contamination. We did not detect specific organisms in the histology, but the clinical appearance and timing would suggest that this was the most likely cause. The graft in this eye did not survive. In the eyes of the second cohort (prednisolone + cyclosporine A) we detected a lower number of Iba1 and CD8-positive cells. This is likely because the surgical grafting procedure (retinotomy and insertion of cannula) inevitably causes the disruption of some blood vessels, enabling the passive infiltration by immune cells and also actively attracting the immune cells and scavenging macrophages to the tissue injury site [128]. Suppression of the innate (antigen independent) immunity is important for xenogeneic graft preservation independent of the immune compatibility [120,70,122], which may be a cause for rapid demise of ocular grafts in some experiments [8,70]. The immunoprivileged ocular space is able to reduce the innate immune responses, but is less effective in suppressing the effector T cells [128]. However, the T cell-mediated immune response is expected to be much faster than the humoral immune response (activated B cells and antibodies). Therefore, we expect that if the cats (Subjects 1 and 2) were sacrificed in 1–2 weeks after the surgical procedure, we would have been able to observe much higher number of CD8-positive CTLs in the grafts, reflecting the fast kinetics of CD8-positive CTL response to antigens [108].

We did not observe any tumorigenesis in cohort 1 (subjects 1 and 2) and cohort 2 (subjects 3, 4, and 5). However, we found the presence of some Ki67-positive cells in the graft, which is expected during retinogenesis [129–131], in line with reports by others [72].

Axonal and synaptic connectivity between the hESC-retinal tissue and recipient degenerating retina are needed to create a functional biological “retinal patch,” which can receive and transmit visual information from PRs of the graft to RGCs of the recipient retina [27]. Our work demonstrated a robust and rapid establishment of initial axonal connectivity (Fig. 5 and Supplementary Figs. S11, Fig. 5 and S12, S13) and initial synaptic connectivity (Fig. 6) between the grafted hESC-retinal tissue from the graft and the recipient cat retina in prednisolone + cyclosporine A-immunosuppressive conditions. The human origin of these projections emanating from the graft was clearly established by using STEM121 antibody, which has been used previously for staining human axons in xenogeneic grafts [8,59,78]. To investigate the extent of maturation of the retinal neurons in grafted retinal organoids, we stained the sections with the antibody to GABA, which is a major inhibitory neurotransmitter in the vertebrate retina [132] and also regulates neuronal differentiation and neural retinal circuit development [133]. We found that the minority of retinal neurons in grafted retinal organoids expressed GABA. We also found GABA in some CALB2-positive cytoplasmic projections connecting the graft and the recipient cat retina (Supplementary Fig. S10).

We observed bidirectional communication between the surviving grafts and the recipient cat retina (Fig. 5). These fibers (projections) connecting the grafts and the host retina were clearly young pathfinding axons, as we demonstrated by staining sections with STEM121 + DCX (Supplementary Fig. S11), and also with pan-axonal cocktail antibody SMI-312 (Supplementary Fig. S12). DCX (a microtubule-associated marker) is present in young neurons and young pathfinding axons [78,112,134–137]), and is a robust marker of early stages of axonal formation [111,112]. Therefore, DCX staining could be expected in young human axons emanating from the grafts [78], especially at the area of the growth cone [135]. In agreement with this, we found DCX-positive, STEM121-positive fibers (Supplementary Fig. S11). However, SMI-312 pan-axonal neurofilament antibody is specific to axons. In addition to finding many SMI-312-positive fibers within the grafts, we found SMI-312-positive fibers connecting the graft and the host, which at least in some cases clearly originated in the cat INL (Supplementary Fig. S12). Interestingly, we also found that some of the STEM121-positive fibers were not ending at RGC layer. but were running parallel to the host RGC layer toward the optic nerve (Supplementary Fig. S13). In earlier work, we showed that bundles of human STEM121[+] axons can travel about 1.2–1.4 mm from the graft in 6 weeks following white matter tracts [78]. In this study, the distance between the edge of the graft and the optic nerve head was between 2 and 3 mm (Supplementary Fig. S13). It is plausible to expect few fast axons emanating from the graft to reach the target 2–3 mm away in about 5.5 weeks.

Collectively, our results indicate that the fibers connecting the cat retina and the grafts are young pathfinding axons and that the connectivity is bidirectional.

We also observed patches of SYP-positive staining in the grafts and in the cat ONL and INL/IPL, and on CALB2-positive fibers (Fig. 6). This matches our previous observation about the ability of retinal neurons in organoids to initiate synaptogenesis [56]. In addition, earlier observations (IN, unpublished observation and [8,78]) point to the expression of SYP marker in young neural grafts in the recipient central nervous system (CNS), where small SYP-positive patches of cells and early synaptic boutons (mostly *boutons en passant*, typical for neural grafts [78,138], and some *terminaux boutons*) can be seen in and around the grafts within a few weeks after grafting.

Collectively, such connectivity, together with a robust immunosuppression protocol, should allow for rapid development of preclinical in vivo work focused on using hESC-retinal tissue for vision restoration in clinically relevant large-eye cat animal models [64,65].

Technologies such as retinal prosthetic devices [139–142] and fetal retina transplantation [15,18,19,38–40,57,143,144] indicate that introducing new functional light-capturing sensors (photosensitive diodes in case of neuroprosthetic devices and photoreceptors in case of fetal retina grafts) is an appropriate way forward in treating RD. However, both of these technologies have limitations. The use of fetal retinal tissue has ethical challenges. Neuroprosthetic devices, however, have their own limitations due to gradual loss of connectivity between neuroprosthetic electronic implant and the neurons of the recipient [145–147]. Higher pixel density and smaller pixel size lead to higher vision resolution [148]. The hESC-derived retinal tissue may be a leap forward from neuroprosthetic devices as functional retinal tissue graft can provide many more light-sensing units (photoreceptors), and therefore may be a path forward toward permanently restoring a much higher resolution of vision.

## Supplementary Material

Supplemental data

Supplemental data

Supplemental data

Supplemental data

Supplemental data

Supplemental data

Supplemental data
