# Supplementary material for: Transplantation of Human Embryonic Stem Cell-Derived Retinal Tissue in the Subretinal Space of the Cat Eye
Source: Stem Cells Dev. 2019 Aug 23;28(17):1151–66. doi: 10.1089/scd.2019.0090 (PMC6708274; doi:10.1089/scd.2019.0090)
Supplement: Supplemental data [file Supp_TableS1.pdf]

Supplementary Table S1. List of Primary Antibodies

Antibody	*Host*	*Source (Cat. No)*	*Dilution*
PAX6	Rabbit	Covance (PRB-278P)	1:500
NEUROD1	Mouse	Santacruz (sc46684)	1:250
CALB2	Rabbit	Abcam (ab702)	1:500
CHX10	Rabbit	GeneTex (GTX114143)	1:500
OTX2	Rabbit	Abcam (ab21990)	1:250
ZO-1	Rabbit	Thermo Fishers (402200)	1:500
BLIMP1	Mouse	Santacruz (sc47332)	1:250
CRX	Rabbit	Genetex (GTX124188)	1:250
BRN3A	Mouse	Santacruz (sc-8429)	1:250
IBA1	Rabbit	Wako (019–19741)	1:500
HNu	Mouse	Abcam (ab191181)	1:500
CD8	Rabbit	Abcam (ab4055)	1:500
STEM121	Mouse	Cellartis (Y40410)	1:500
Human synaptophysin (SYP)	Mouse	Thermo Fisher (14-6525-82)	1:500
KU80	Rabbit	Abcam (ab80592)	1:500
DCX	Guinea pig	Millipore (Ab2253)	1:500
GABA	Guinea pig	Abcam (ab17413)	1:500
PMEL17	Rabbit	Abcam (ab137062)	1:250
NF-H	Rabbit	Millipore (AB1989)	1:500
SMI-312	Mouse	Biolegend (837904)	1:500
