# Supplementary material for: Transplantation of Human Embryonic Stem Cell-Derived Retinal Tissue in the Subretinal Space of the Cat Eye
Source: Stem Cells Dev. 2019 Aug 23;28(17):1151–66. doi: 10.1089/scd.2019.0090 (PMC6708274; doi:10.1089/scd.2019.0090)
Supplement: Supplemental data [file Supp_FigureS1-S2.pdf]

Supplementary Data

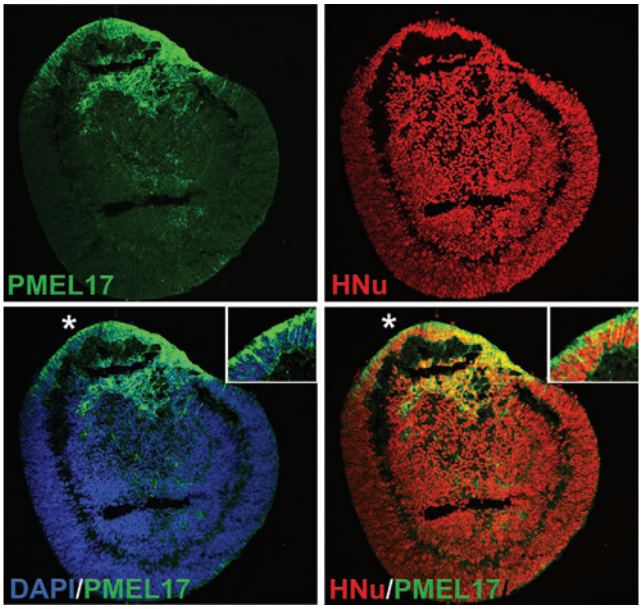

SUPPLEMENTARY FIG. S1. Immunostaining the retinal organoid with pigmented RPE marker PMEL17 shows patches of retinal organoids were pigmented. HNu stains the human nuclei. The *insets* are high magnification of area marked with *asterisk* (*). DAPI counterstains nuclei.

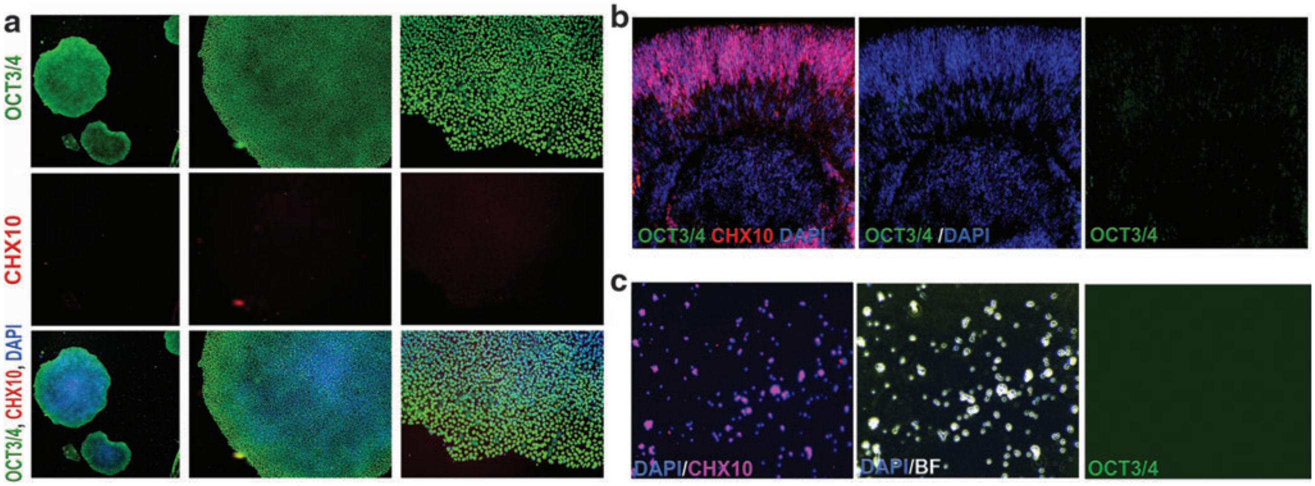

SUPPLEMENTARY FIG. S2. Immunolabeling human ES colonies and retinal organoids with antibodies to OCT3/4 and CHX10. **(a)** HES3 colonies immunostained with human pluripotent marker OCT3/4 and pan-neural retinal progenitor marker CHX10 show presence of OCT3/4 and absence of CHX10. DAPI counterstains nuclei. **(b)** Immunostaining the retinal organoid sections with CHX10 and OCT3/4 shows absence of OCT3/4 in the retinal organoids and presence of CHX10. DAPI counterstains nuclei. **(c)** Immunostaining the dissociated retinal organoids confirms the presence of CHX10 and absence of OCT3/4. DAPI counterstains nuclei.
