# Supplementary material for: Transplantation of Human Embryonic Stem Cell-Derived Retinal Tissue in the Subretinal Space of the Cat Eye
Source: Stem Cells Dev. 2019 Aug 23;28(17):1151–66. doi: 10.1089/scd.2019.0090 (PMC6708274; doi:10.1089/scd.2019.0090)
Supplement: Supplemental data [file Supp_FigureS3-S4.pdf]

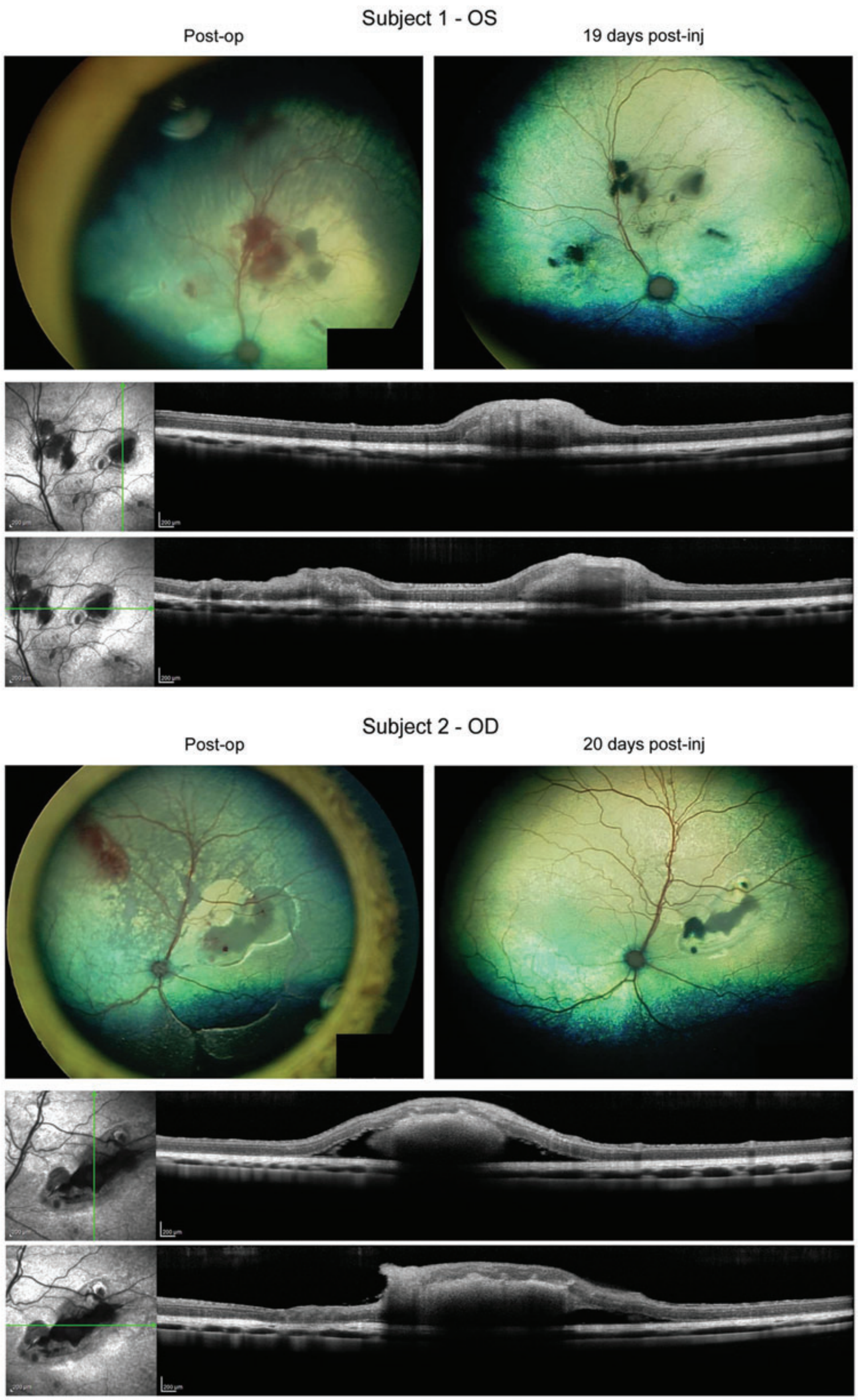

SUPPLEMENTARY FIG. S3. RetCam fundus images, cSLO fundus images and high-resolution retinal cross-section SD-OCT images of subretinal grafts at different time points in subjects 1–5. Retinal organoids are present in the subretinal space between 19 and 21 days postinjection on those images. The grafts are visible both on RetCam images (*top row*) and SD-OCT images (*bottom rows*). SD-OCT, spectral-domain optical coherence tomography; cSLO, scanning laser ophthalmoscope.

(*continued*)

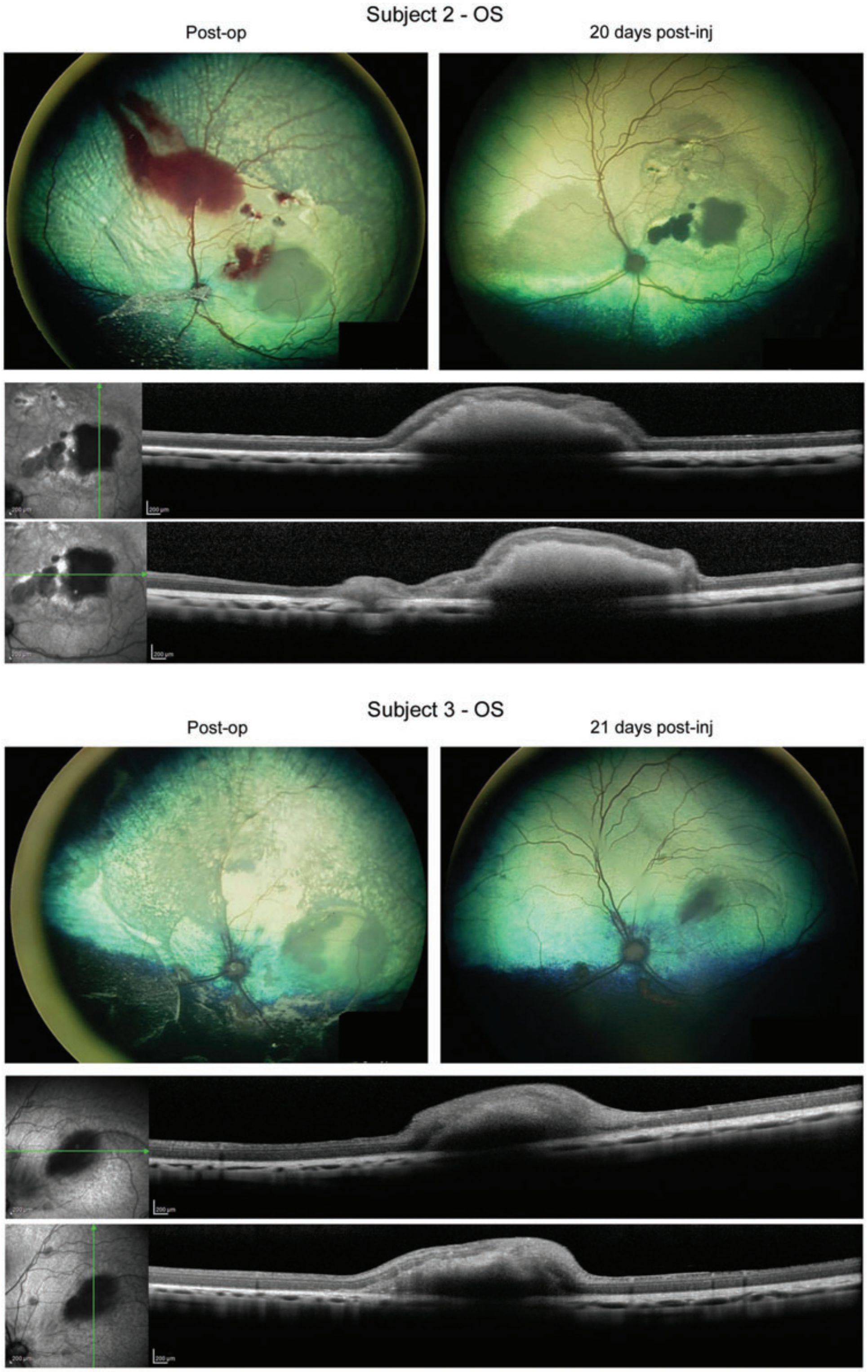

SUPPLEMENTARY FIG. S3. (Continued).

(*continued*)

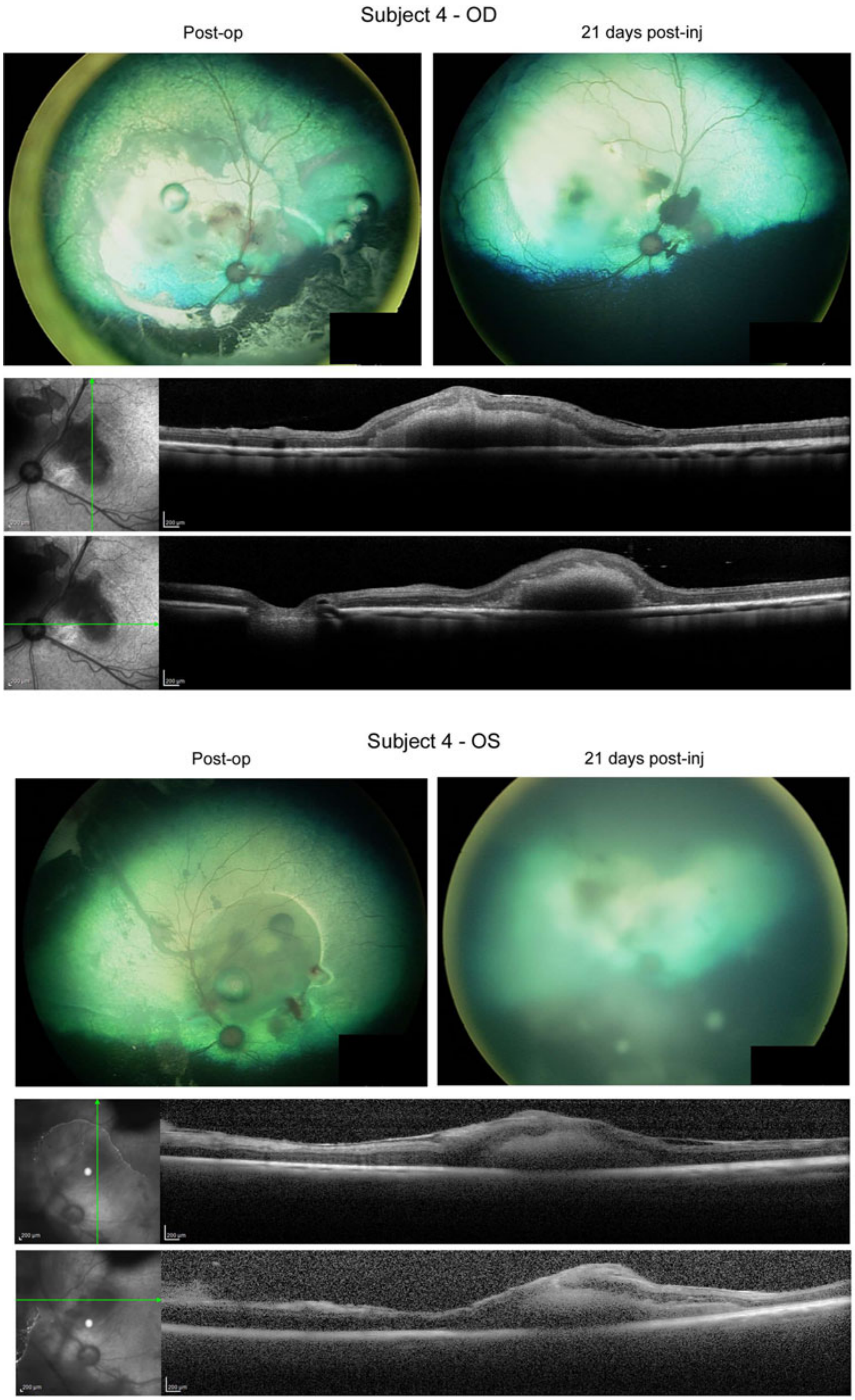

SUPPLEMENTARY FIG. S3. (Continued).

(*continued*)

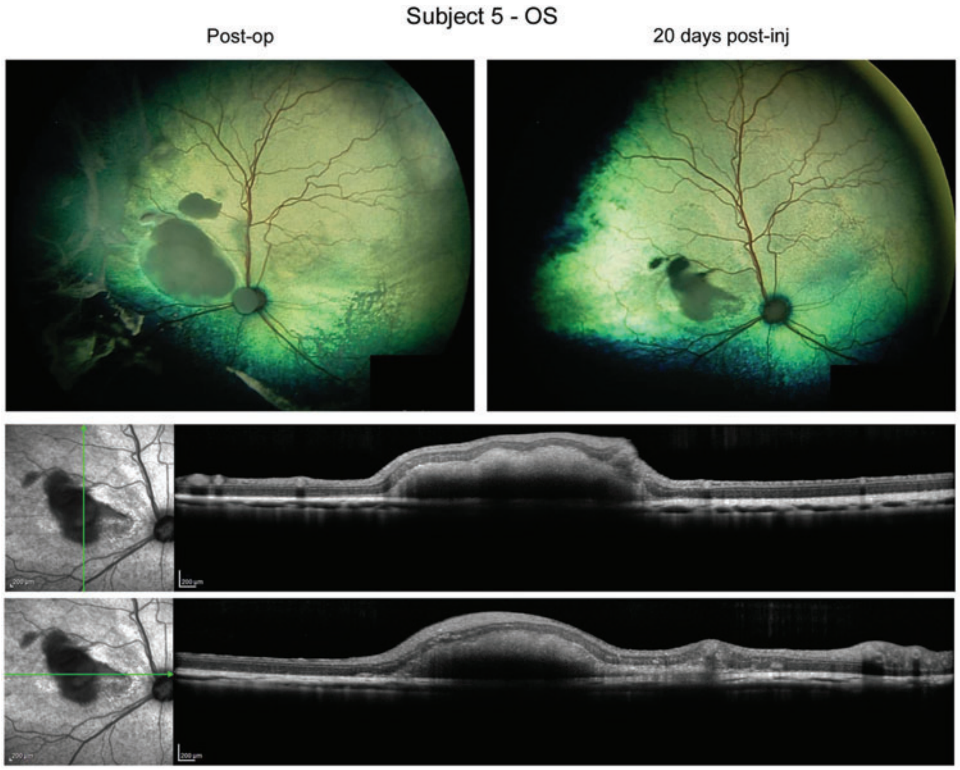

SUPPLEMENTARY FIG. S3. (Continued).

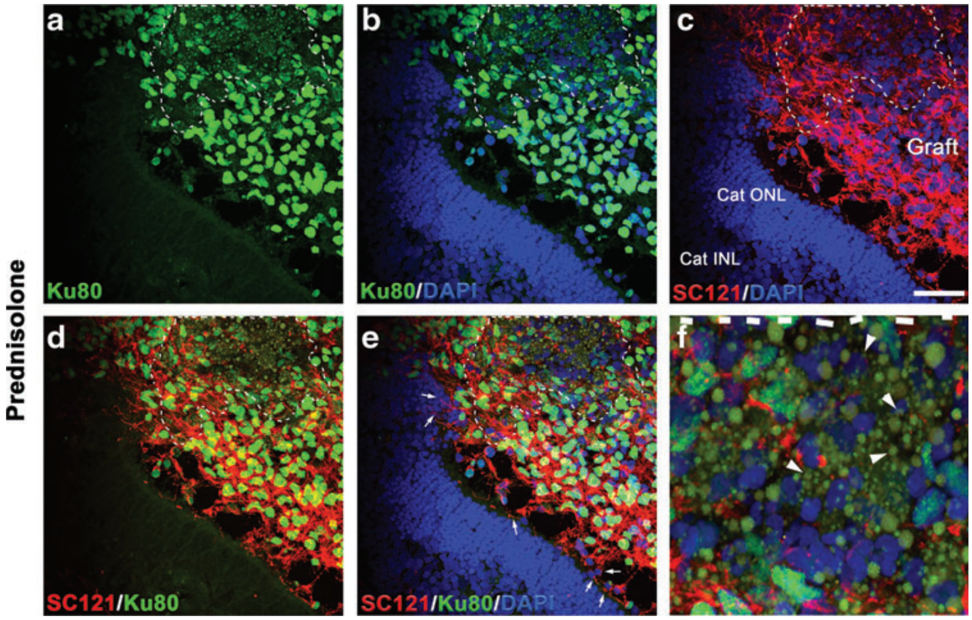

SUPPLEMENTARY FIG. S4. Small patches of surviving human nuclei in subject 2 maintained with prednisolone immunosuppression. **(a–e)** show immunostaining of the graft with human nuclei marker Ku80 and human cytoplasmic marker STEM121. Arrow in panel **e** indicates SC121 positive fibers making connectivity with the cat ONL. **(f)** is magnification of the area marked with *dotted line* showing fragmentation of nuclei (shown with *arrow heads*) and lack of STEM121 staining in the graft. DAPI counter stains the nuclei. Scale bar: 50 μm.
