# Supplementary material for: Transplantation of Human Embryonic Stem Cell-Derived Retinal Tissue in the Subretinal Space of the Cat Eye
Source: Stem Cells Dev. 2019 Aug 23;28(17):1151–66. doi: 10.1089/scd.2019.0090 (PMC6708274; doi:10.1089/scd.2019.0090)
Supplement: Supplemental data [file Supp_FigureS5-S7.pdf]

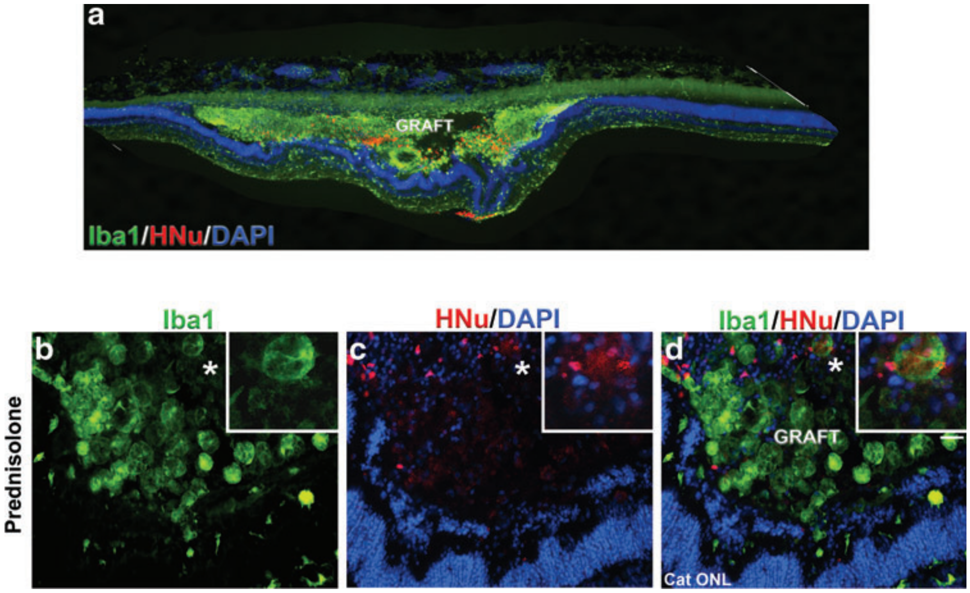

SUPPLEMENTARY FIG. S5. Immunohistochemical localization of Iba1 in subretinal graft of subject 2 (prednisolone only) maintained for 66 days. **(a)** Composite image shows the graft full of Iba1-positive cells. **(b)** High magnification image showing Iba1-positive macrophages. **(c)** High magnification image showing fragmented HNu-positive nuclei. **(d)** Iba1-positive macrophages engulfing human grafts [stained with HNu]. *Insets* show area marked with *asterisks*.

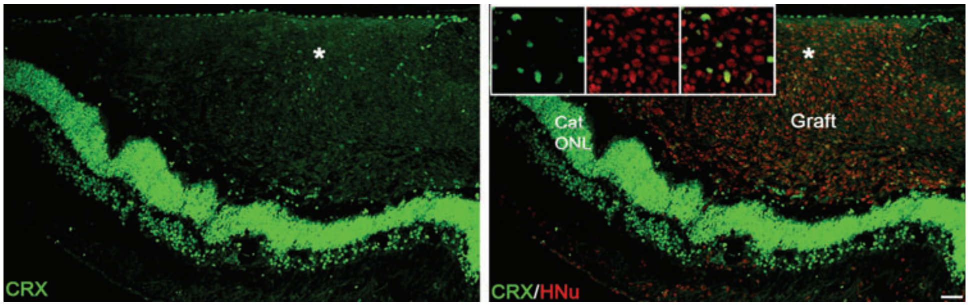

SUPPLEMENTARY FIG. S6. Presence of CRX-positive cells in the graft and host tissue. *Inset* show magnification of the area marked with *asterisks*.

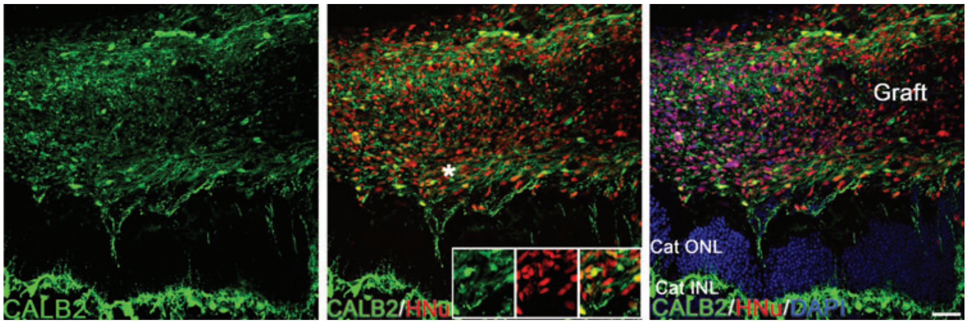

SUPPLEMENTARY FIG. S7. Presence of CALB2- and HNu-positive cells in the subretinal grafts. *Inset* shows magnification of the area marked with *asterisks*.
