# Supplementary material for: Transplantation of Human Embryonic Stem Cell-Derived Retinal Tissue in the Subretinal Space of the Cat Eye
Source: Stem Cells Dev. 2019 Aug 23;28(17):1151–66. doi: 10.1089/scd.2019.0090 (PMC6708274; doi:10.1089/scd.2019.0090)
Supplement: Supplemental data [file Supp_FigureS8-S10.pdf]

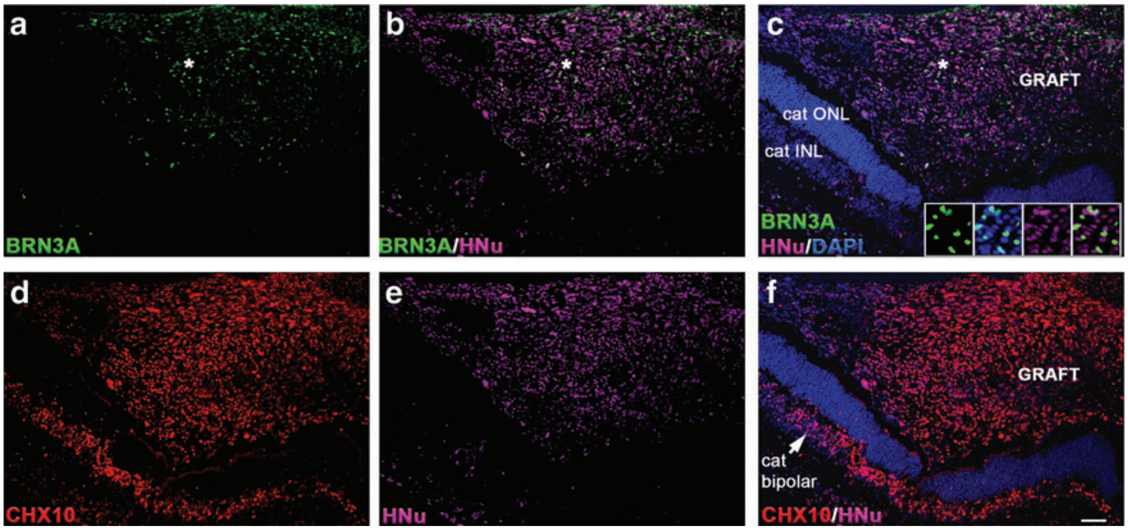

SUPPLEMENTARY FIG. S8. Presence of retinal ganglion cell marker (BRN3A) and neural retina progenitor marker CHX10 in the graft and host tissue. **(a–f)** *Inset* in panel **c** is the magnification of area marked with *asterisk*. CHX10 staining was also seen in bipolar cells of cat retina (*arrow*). HNu staining showing presence of human graft.

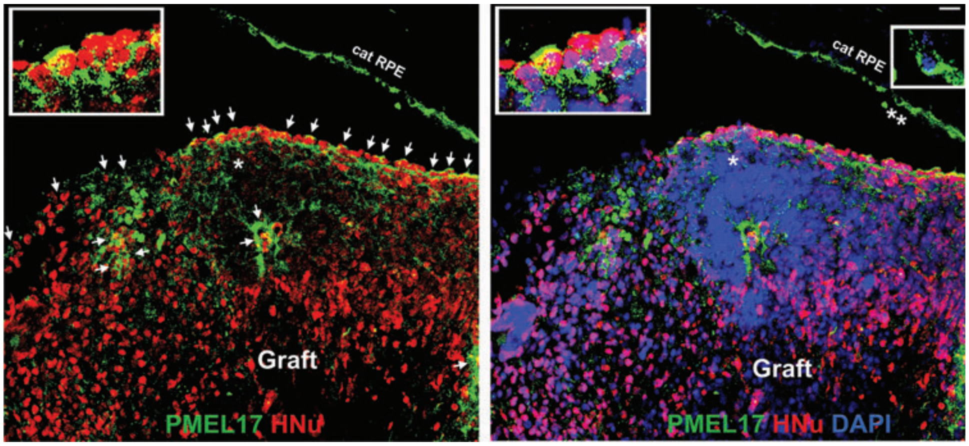

SUPPLEMENTARY FIG. S9. Subretinal graft immunostained with PMEL17 reveals the presence of pigmented RPE in the graft. HNu stains the human nuclei. The *insets* are high magnification of area marked with *asterisk* (* and **). *White arrows* point to graft-derived human RPE cells stained with PMEL17 antibody. DAPI counterstains nuclei. Scale bar: 20 μm.

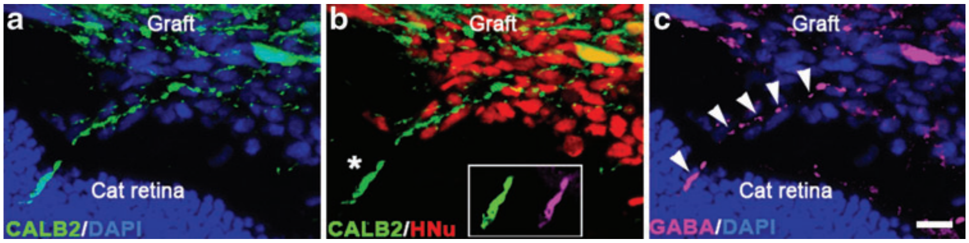

SUPPLEMENTARY FIG. S10. Co-immunolabeling of the junction between the graft and the host with CALB2, HNu, and GABA **(a–c)** shows some CALB2-positive and GABA-positive projections emanating from the graft to the host (*white arrow*). *Asterisk* (*) shows the area magnified in the *inset*. Scale bar: 50 μm.
