# Supplementary material for: Transplantation of Human Embryonic Stem Cell-Derived Retinal Tissue in the Subretinal Space of the Cat Eye
Source: Stem Cells Dev. 2019 Aug 23;28(17):1151–66. doi: 10.1089/scd.2019.0090 (PMC6708274; doi:10.1089/scd.2019.0090)
Supplement: Supplemental data [file Supp_FigureS11-S12.pdf]

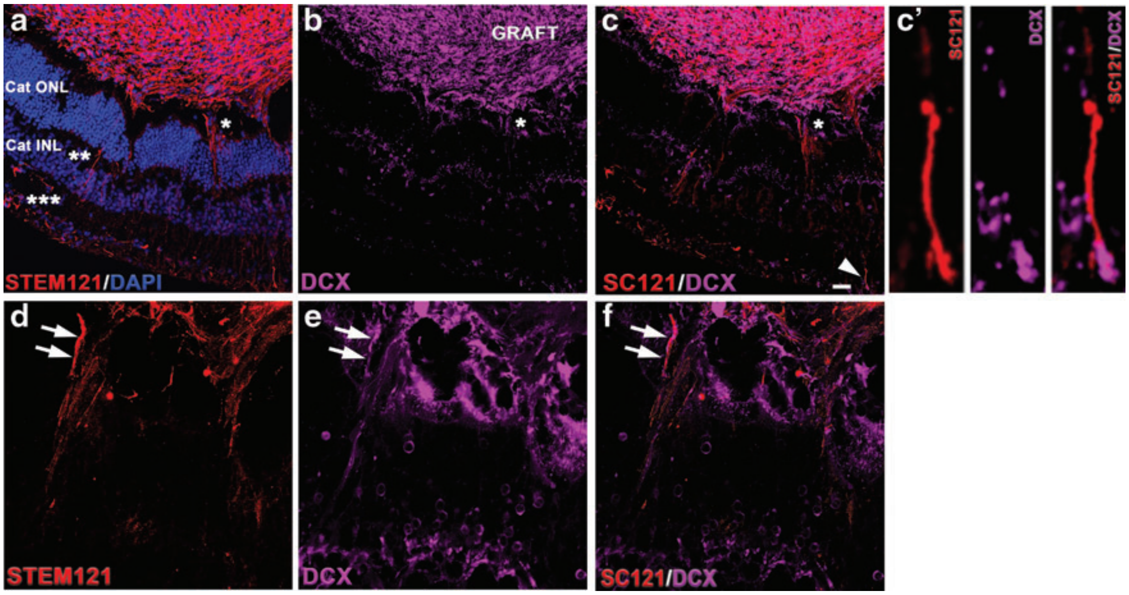

SUPPLEMENTARY FIG. S11. Axonal connectivity between the grafts and the recipient cat retina. Immunolocalization shows presence of human cytoplasmic marker (STEM121) and microtubule-associated marker DCX in the graft. *Asterisk* (*) in **(a–c)** are projections emanating from the graft. **(d–f)** are magnified images of the area marked with *asterisks* in **(a–c)**, respectively. *Arrow* shows the projection emanating from the graft overlapped between STEM121 and DCX. **(c')** is magnification of **(c)** (*arrowhead*) showing extension of STEM121- and DCX-positive projections into the RGC layer of the host.

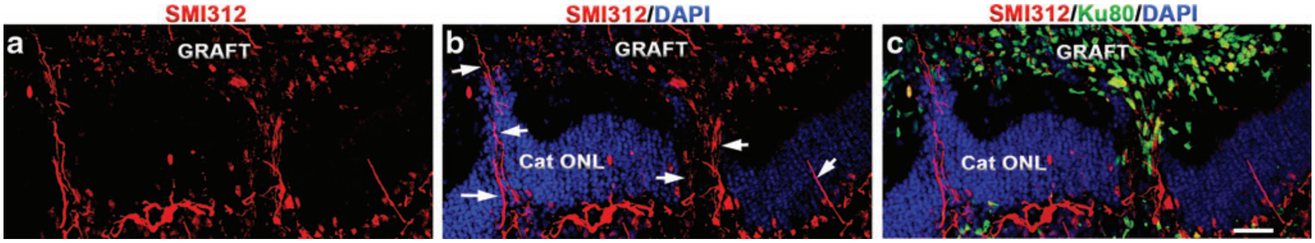

SUPPLEMENTARY FIG. S12. Immunostaining the graft with pan-axonal neurofilament marker SMI312, human marker Ku80 shows axons connecting the graft and host retina **(a–c)**. DAPI counter stains the nuclei. Scale bar: 50 µm.
