# Supplementary material for: Transplantation of Human Embryonic Stem Cell-Derived Retinal Tissue in the Subretinal Space of the Cat Eye
Source: Stem Cells Dev. 2019 Aug 23;28(17):1151–66. doi: 10.1089/scd.2019.0090 (PMC6708274; doi:10.1089/scd.2019.0090)
Supplement: Supplemental data [file Supp_FigureS13.pdf]

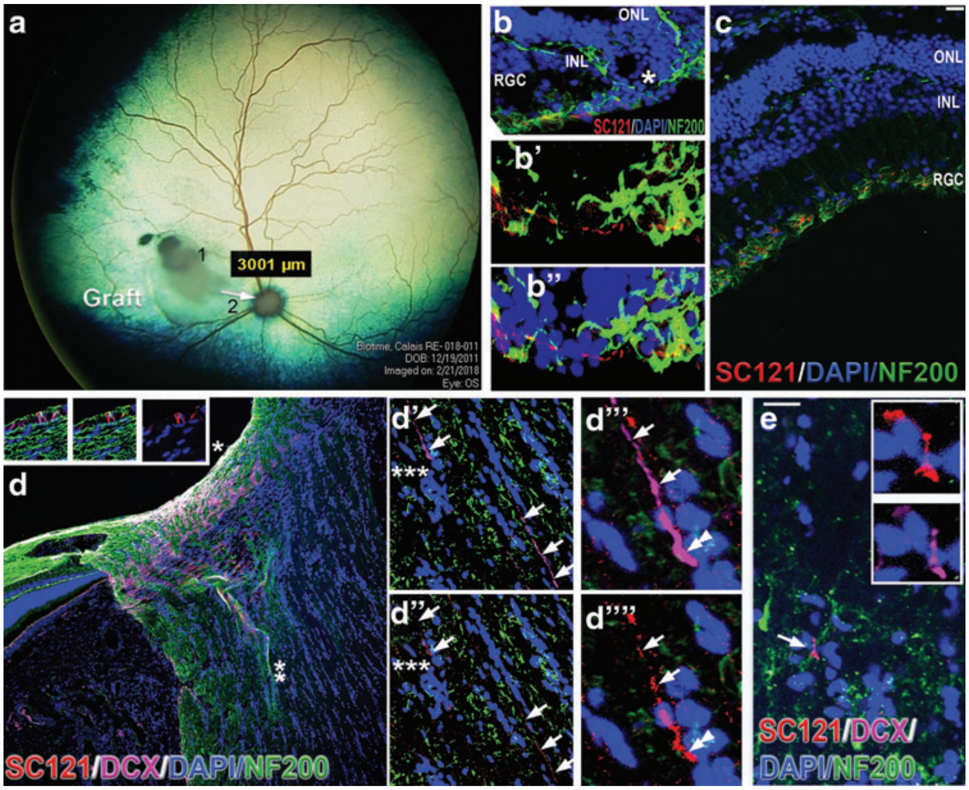

SUPPLEMENTARY FIG. S13. Human STEM121-positive fibers in the optic nerve. **(a)** RetCam fundus image shows the distance between the graft and host optic nerve. **(b)** Overview of the cat retina. This is a single optical section in the area marked '1' in panel 'a' showing SC121 and NF200 staining in CAT RGC layer. The *asterisk* marks the area **(b)** enlarged in the two *insets* **(b', b'')** showing SC121-positive fiber is negative for NF200. **(c)** The SC121 [+] fibers in the host RGC layer in the area marked ''2'' in **(a)**. Scale bar: 20 µm. **(d)** In the optic nerve head, few SC121 [+] fibers are present (*), and they overlap with DCX. The *inset* is the enlargement of the area shown in the *asterisk* in **(d)**. The area marked with *double asterisk* (**) is enlarged in **(d', d'')** (which show human fibers, *white arrows*, positive for SC121 and for DCX, but negative for NF200). The orientation is the same as in the main **(d)** (the tips of the fibers point away from the optic nerve head and into the optic nerve). The areas in **(d', d'')** marked with *triple asterisks* are further enlarged in the **(d''', d'''')**, respectively, to focus on a single tip of a SC121[+] [DCX]+ fiber, which looks like a growth cone (*double arrowheads*); **(e)** Area in the optic nerve head, where SC121[+] human fiber (also positive for DCX, but negative for NF200) was found. Scale bar: 20 µm. DCX, doublecortin.
